# Outcomes of EUS-guided gallbladder drainage in malignant distal biliary obstruction: a systematic review and meta-analysis

**DOI:** 10.1016/j.igie.2023.07.003

**Published:** 2023-07-14

**Authors:** Karim T. Osman, Ahmed M. Abdelfattah, Maisa E. Elbadawi, Tarek Nayfeh, Dhruval Amin, Lina Elkhabiry, Carol Spencer, Prashanth Rau, Neil Marya

**Affiliations:** 1 Division of Gastroenterology; 3 Department of Internal Medicine; 7 Department of Library Services, Lahey Hospital and Medical Center, Burlington, MA; 2 Division of Gastroenterology; 5 Department of Internal Medicine, UMass Chan Medical School, Worcester, MA; 4 Evidence-based Practice Center, Mayo Clinic, Rochester, MN; 6 Department of Internal Medicine, University of Alexandria, Alexandria, Egypt

## Abstract

**Background and aims:**

EUS-guided gallbladder drainage (EUS-GBD) has been described as an alternative palliative treatment for malignant distal biliary obstruction (MDBO). This study assessed the outcomes of EUS-GBD for MDBO.

**Methods:**

We conducted a comprehensive literature review of the MEDLINE, Embase, Cochrane Central Register of Controlled Trials, Cochrane Database of Systematic Reviews, and Scopus databases. Formal meta-analysis of pooled outcomes of EUS-GBD for MDBO was performed by using a random effects model. Outcomes of interest were technical success, clinical success, adverse events, and reintervention rates. Pooled estimates were calculated following the restricted maximum likelihood method using a random effects model, and heterogeneity was assessed by using the *I*^2^ statistic.

**Results:**

Fifteen unique articles (a total of 161 patients) were included in the meta-analysis. The pooled technical success rate was 92.10% (95% confidence interval [CI], 84.70-96.09), and the pooled clinical success rate was 81.62% (95% CI, 74.27-97.24). The pooled reintervention rate after achieving clinical success was 13.02% (95% CI, 8.02-20.43), and the pooled adverse event rate was 13.81% (95% CI, 9.19-20.23). The *I*^2^ was 0 for all meta-analyses.

**Conclusions:**

This meta-analysis is the first to evaluate outcomes of EUS-GBD in MDBO. We report high technical and clinical success with relatively low reintervention and adverse event rates. There was no heterogeneity in the data. EUS-GBD is a feasible palliative option for MDBO when conducted at experienced centers.

Malignant distal biliary obstruction (MDBO) is typically managed with ERCP. However, in patients with MDBO, duodenal or periampullary invasion, distal biliary obstruction, or altered anatomy from prior surgeries can make ERCP challenging. Clinicians typically pursue EUS-guided bile duct drainage (EUS-BD) if ERCP failed or is not feasible.^1^ However, EUS-BD can fail for various reasons, including inability to access the bile duct via the stent, inability to pass the guidewire, small bile duct diameter, and lack of a vessel-free window.^2^, ^3^, ^4^, ^5^ Our interventional radiology colleagues have performed other minimally invasive interventions such as percutaneous transhepatic biliary drainage (PTBD) to treat MDBO.^6^^,^^7^ However, PTBD has been associated with high rates of morbidity and adverse events.^3^

In 2013, Itoi et al^4^ described the first report of EUS-guided gallbladder drainage (EUS-GBD) as a palliative approach for MDBO. Since then, EUS-GBD has become increasingly used when ERCP and EUS-BD fail. However, most of the literature is documented in sporadic case reports and case series. The success rates and adverse events of EUS-GBD in this patient population have not been reported, which poses a challenge for both clinicians and patients to make an educated judgment on the pros and cons of pursuing EUS-GBD compared with ERCP, EUS-BD, and PTBD.

We therefore conducted a meta-analysis to assess the technical success, clinical success, adverse events, and reintervention rates of EUS-GBD in MDBO.

## Methods

The systematic review is reported according to the Preferred Reporting Items for Systematic Reviews and Meta-Analyses statement.^8^

### Search strategy

A comprehensive search of several databases from each database's inception to March 15, 2023 (English language only), was conducted. The databases included MEDLINE, Embase, Cochrane Central Register of Controlled Trials, Cochrane Database of Systematic Reviews, and Scopus. The search strategy was designed and conducted by an experienced librarian (C.S.) with input from the study’s principal investigator. The search strategy is reported in Supplementary Table 1 (available online at www.igiejournal.org).

### Eligibility criteria

Studies included in the systematic review, whether abstracts or full-length manuscripts, met the following inclusion criteria: (1) patients: adults, age >18 years, with obstructive jaundice secondary to unresectable MDBO, (2) intervention: EUS-GBD, and (3) outcomes: technical success, clinical success, reintervention rates, and adverse events.

### Study selection process

Three independent reviewers (K.T.O., A.M.A., and M.E.E.) reviewed the titles and abstracts of all citations identified by the database search. Full-text manuscripts were retrieved for the included references and were subsequently screened for eligibility by 3 independent reviewers (K.T.O., A.M.A., and M.E.E.). Disagreements at this level were resolved by consensus.

### Data extraction

Data regarding the baseline characteristics, sample size, and outcomes of interest were abstracted independently onto a standardized form by 2 reviewers (K.T.O. and A.M.A.). Where necessary, we attempted to contact the corresponding authors of individual studies to obtain additional details of interventions and outcomes. Technical success was defined as successful stent deployment. Clinical success was defined as one of the following: (1) decrease in serum bilirubin >50%, (2) normalization of serum bilirubin, or (3) improvement in jaundice/symptoms. Reintervention was defined as the need for reintervention after achieving clinical success. Adverse events were classified as immediate and delayed adverse events. Immediate adverse events were defined as adverse events that occurred intraprocedurally until the first 24 hours after the procedure. Delayed adverse events were defined as adverse events that occurred after the first 24 hours of performing the EUS-GBD.

### Quality assessment

The quality of individual case reports was assessed by using the methodologic assessment tool described by Murad et al.^9^ This tool evaluates 4 domains (Selection, Ascertainment, Causality, and Reporting) by answering certain questions for each domain. The question evaluating Selection was “Does the patient represent the whole experience of the investigator (center) or is the selection method unclear to the extent that other patients with similar presentation may not have been reported?” For the domain of Ascertainment, was the outcome reliably ascertained? For the domain of Causality, was follow-up adequate? Adequate follow-up was defined by the authors of this study as being 2 weeks. For the domain of Reporting, was the case described with sufficient details to allow other investigators to replicate the research or to allow practitioners to make inferences related to their own practice?

The quality of individual retrospective cohorts was assessed by using the Newcastle-Ottawa Scale.^10^ This tool evaluates 2 domains (Selection and Outcome). The question evaluating Selection was: “was the cohort representative of the whole experience of the investigator (center) or is the selection method unclear to the extent that other patients with similar presentation may not have been reported?” Three questions evaluated the domain of Outcome: “was the outcome reliably ascertained?”; “was follow-up period adequate?”; and “were all subjects accounted for at the end of the follow-up period?”

All questions are scored 0 if answered “No” and 1 if answered “Yes.” Disagreements in answering questions were resolved by consensus. For each individual study, a total score of 4, 3, and <3 classified the study as “good quality,” “fair quality,” and “poor quality,” respectively.

### Data synthesis

Analysis was performed by using R version 4.0.4 (R Foundation for Statistical Computing, Vienna, Austria). Pooled estimates of outcomes were calculated following the methods suggested by Raudenbush and Bryk^11^ using the random effects model. Because a random effects model accounts for variance from both within the study and between different studies, it reports an average estimate; as such, we reported the 95% confidence interval (CI) as well.^12^^,^^13^ Heterogeneity was assessed by using the *I*^2^ statistic. *I*^2^ values <30%, 30% to 60%, 61% to 75%, and >75% were indicative of low, moderate, substantial, and considerable heterogeneity, respectively.

If any outcome had more than a low level of heterogeneity, we conducted a meta-analysis of proportions after excluding one study at a time to identify studies that are influential on the overall results of each outcome. This was done by using the R package metafor.^14^^,^^15^ Sensitivity analysis was then conducted after excluding those studies.

### Publication bias

Publication bias was ascertained qualitatively by visual inspection of funnel plots and quantitively by using Egger’s regression test.^16^, ^17^, ^18^

### Meta-regression model

Random effect meta-regression was used to assess the association between the percentage of patients who had lumen-apposing metal stents (LAMSs) (independent variable) and the proportion of outcomes. Meta-regression results were expressed as a regression coefficient, 95% CI, and a *P* value.^19^ The regression coefficient quantifies magnitude of change in the Freeman-Tukey double arcsine transformation of the proportion of outcomes of interest associated with a 1% increase in the percentage of having LAMSs placed. A value of 0 denotes no relationship between the variables, whereas –1 or 1 conveys a maximum negative or positive relationship, respectively.

## Results

**Supplementary Figure 1:**
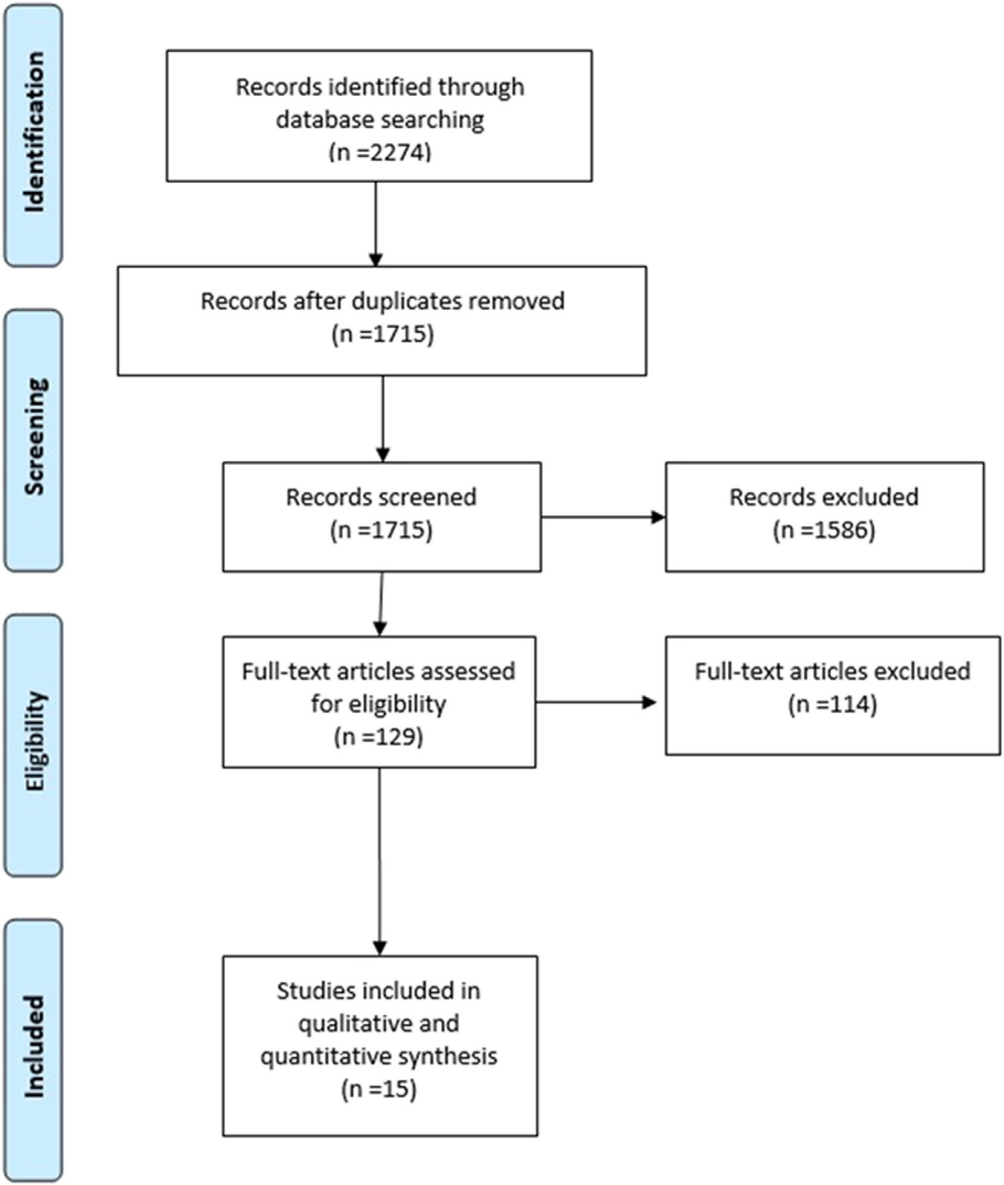
Flowchart of included studies.

After excluding duplicates, a total of 1597 articles were identified and screened for eligibility; of these, 120 full-text articles were retrieved and subsequently evaluated for inclusion. Fifteen studies^2^, ^3^, ^4^, ^5^^,^^20^, ^21^, ^22^, ^23^, ^24^, ^25^, ^26^, ^27^, ^28^, ^29^, ^30^ were finally included. Supplementary Figure 1 (available online at www.igiejournal.org) shows the process of identifying relevant studies.

### Population characteristics and quality assessment of included articles

Table 1 summarizes the study and population characteristics. A total of 161 patients were included, with a median follow-up of 4.25 (2.40-10.50) months. The median age of patients when they underwent EUS-GBD was 67.65 (62.33-71.08) years. Most of the patients were male (54.95%). The most common malignancy was pancreatic cancer (63.06%), followed by cholangiocarcinoma (8.11%), followed by ampullary or duodenal adenocarcinoma (4.50%). EUS-GBD was pursued in all studies either due to failed ERCP/EUS-BD or as first-line therapy in anticipated failure of former procedures due to poor anatomy. Cystic duct anatomy was reviewed in 12 studies^2^, ^3^, ^4^, ^5^^,^^21^, ^22^, ^23^, ^24^^,^^27^, ^28^, ^29^, ^30^ before EUS-GBD; this anatomy review was not mentioned in the remaining 3 studies.^20^^,^^25^^,^^26^ LAMSs were used in 128 patients and in most of the studies.^2^, ^3^, ^4^, ^5^^,^^20^, ^21^, ^22^, ^23^^,^^25^, ^26^, ^27^, ^28^ Of the studies that used LAMSs, 9 patients had noncautery LAMSs, and 112 patients had electrocautery-enhanced LAMSs; 1 study did not report whether electrocautery was used.^27^ Self-expandable metal stents were used in 15 patients and in only 3 studies.^3^^,^^24^^,^^29^ One study did not report the type of stent placed.^30^

**Table 1:** Study characteristics

Study (year); location	Time period	Sample size (n)	Age, y∗	Sex	Type of malignancy	Site of stent	Type of stent	Follow-up period, mo∗
Rai et al^5^ (2014); India	NR	1	30	M	Ampullary cancer	Stomach	NAGI 14 × 20 mm†	1
Itoi et al^4^ (2013); United States	NR	1	57	M	Head of the pancreas malignancy	Stomach	AXIOS 10 × 10 mm†	12
Chin et al^2^ (2020); New Zealand	August 2016-July 2020	4	NR	NR	NR	NR	Hot AXIOS‡	NR
Pleasant et al^28^ (2020); United States	NR	1	88	M	Ampullary cancer	NR	Hot AXIOS 15 × 10 mm‡	.25
Flor de Lima et al^23^ (2021); Portugal	NR	1	60	F	Cholangiocarcinoma	Stomach	Hot AXIOS 15 × 10 mm‡	2
Paleti et al^27^ (2019); United States	October 2016-November 2018	7	67 ± 13.3	5 M, 2 F	Head of the pancreas malignancy	NR	LAMSs	NR
Issa et al^3^ (2021); United States	2014-2019	28	68 ± 13	16M, 12 F	NR	Duodenum (n = 15), stomach (n = 13)	LAMSs (n = 26), SEMS (n = 2)§	33 (range, 3-64)
Cecinato et al^21^ (2017); Italy	NR	1	81	M	Head of the pancreas malignancy	Jejunal roux limb	Hot AXIOS 10 × 10 mm‡	NR
Lambin et al^25^ (2021); France	July 2016-July 2020	28	NR	NR	Pancreatic cancer (n = 19), cholangiocarcinoma (n = 4), other malignancies (n = 5)	NR	LAMSs‡	3.6 ± 5
Suzuki et al^29^ (2018); Japan	NR	1	70	F	Pancreatic cancer	Duodenum	WallFlex	17
Ligresti et al^26^ (2019); Italy	NR	1	70	F	Adenocarcinoma involving distal CBD and duodenum	Stomach	AXIOS 8 × 8 mm‡	NR
Binda et al^20^ (2021); Italy	June 2015-June 2020	48	74.3 ± 11.7	23M, 25 F	Pancreatic cancer (n = 40), cholangiocarcinoma (n = 2), duodenal and ampullary cancers (n = 2); other malignancies (n = 4)	Duodenum (n = 20), stomach (n = 28)	LAMSs 16 × 30 mm (n = 1), 15 × 10 mm (n = 2), 10 × 10 mm (n = 34), 8 × 10 mm (n = 10) and 6 × 8 mm (n = 1)‖	4.07 ± 5.37
Imai et al^24^ (2016); Japan	January 2006-October 2014	12	67.3 ± 13.9	8 M, 4 F	Pancreatic cancer (n=6), lymph node metastasis (n = 3), cholangiocarcinoma (n = 2), lymphoma (n = 1)	Duodenum (n = 5), stomach (n = 7)	WallFlex 8 × 6 mm	NR
Chang et al^22^ (2019); United States	October 2016-December 2017	9	63.1 (mean)	5 M, 4 F	Pancreatic cancer	Duodenum (n = 5), stomach (n = 4)	AXIOS 15 × 10 mm (n = 6) and 10 × 10 mm (n = 3)‡	4.36
Mangiavillano et al^30^ (2022); Italy	January 2021-May 2021	18	NR	NR	NR	Duodenum (n = 4), stomach (n = 14)	NR	6

The manufacturer information for the stents mentioned in the table is as follows: NAGI, Taewoong-Medical Co; and AXIOS and WallFlex, Boston Scientific.

*NR*, Not reported; *M*, male; F*,* female; *LAMSs*, lumen-apposing metal stents; *SEMSs*, self-expandable metal stents; *CBD,* common bile duct.

∗ Studies with sample size of >1 patient reported values as mean ± standard deviation unless otherwise specified in table.

† Noncautery LAMSs.

‡ Electrocautery-enhanced LAMSs.

§ Twenty patients had electrocautery-enhanced LAMSs, and 6 patients had noncautery LAMSs.

‖ Forty-seven patients had electrocautery-enhanced LAMSs, and 1 patient had noncautery LAMSs.

Twelve articles^2^, ^3^, ^4^, ^5^^,^^21^, ^22^, ^23^, ^24^^,^^26^^,^^29^^,^^30^ were full-length manuscripts, and 3 articles^20^^,^^25^^,^^28^ were abstracts. Eight of the articles were retrospective cohorts,^2^^,^^3^^,^^20^^,^^22^^,^^24^^,^^25^^,^^27^^,^^31^ one article was a prospective study,^30^ and the other half were case reports.^4^^,^^5^^,^^21^^,^^23^^,^^26^^,^^28^^,^^29^ All articles were single-center studies except for 3 studies, which were multicentric.^3^^,^^20^^,^^30^ Three studies were of good quality,^3^^,^^22^^,^^30^ 8 studies were of fair quality,^4^^,^^5^^,^^20^^,^^23^, ^24^, ^25^^,^^27^^,^^29^ and 4 studies were of poor quality^2^,^21^^,^^26^^,^^28^ (Supplementary Table 2, available online at www.igiejournal.org).

### Technical and clinical success

**Figure 1:**
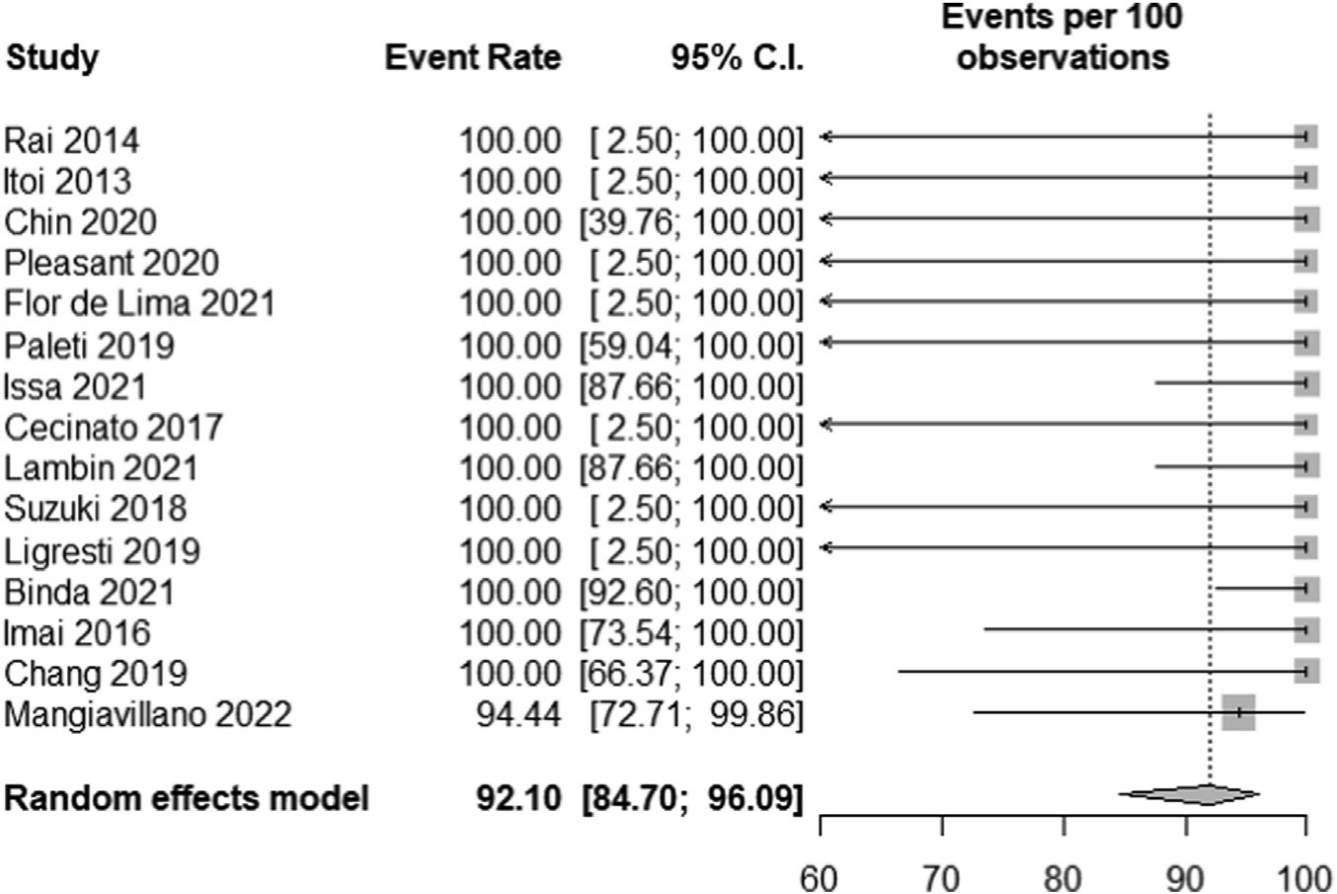
Pooled technical success rate. *CI*, Confidence interval.

All studies reported technical success.^2^, ^3^, ^4^, ^5^^,^^20^, ^21^, ^22^, ^23^, ^24^, ^25^, ^26^, ^27^, ^28^, ^29^, ^30^ One patient did not achieve technical success due to misplacement of the distal flange of the stent; however, this was rescued by immediate placement of another stent.^30^ The pooled technical success rate was 92.10% (95% CI, 84.70-96.09; *I*^2^ = 0) (Fig. 1).

**Figure 2:**
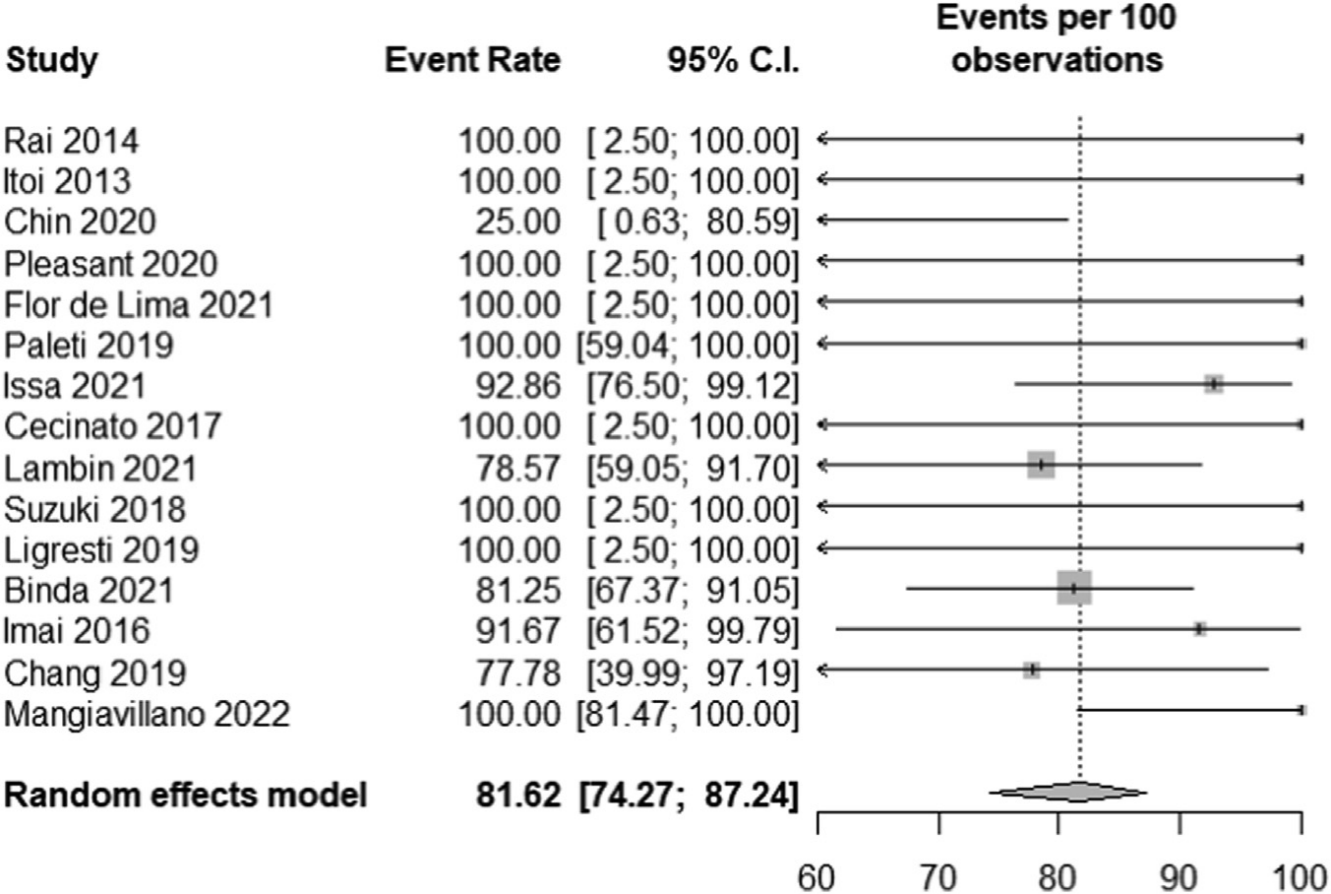
Pooled clinical success rate. *CI*, Confidence interval.

All studies^2^, ^3^, ^4^, ^5^^,^^20^, ^21^, ^22^, ^23^, ^24^, ^25^, ^26^, ^27^, ^28^, ^29^, ^30^ reported clinical success, with a total of 161 patients included in the meta-analysis. The mean reduction in serum bilirubin was 8.80 (6.78-14.02) mg/dL. None of the studies reported change in common biliary duct diameter. The pooled clinical success rate was 81.62% (95% CI, 74.27-97.24; *I*^2^ = 0) (Fig. 2).

### Adverse events

**Figure 3:**
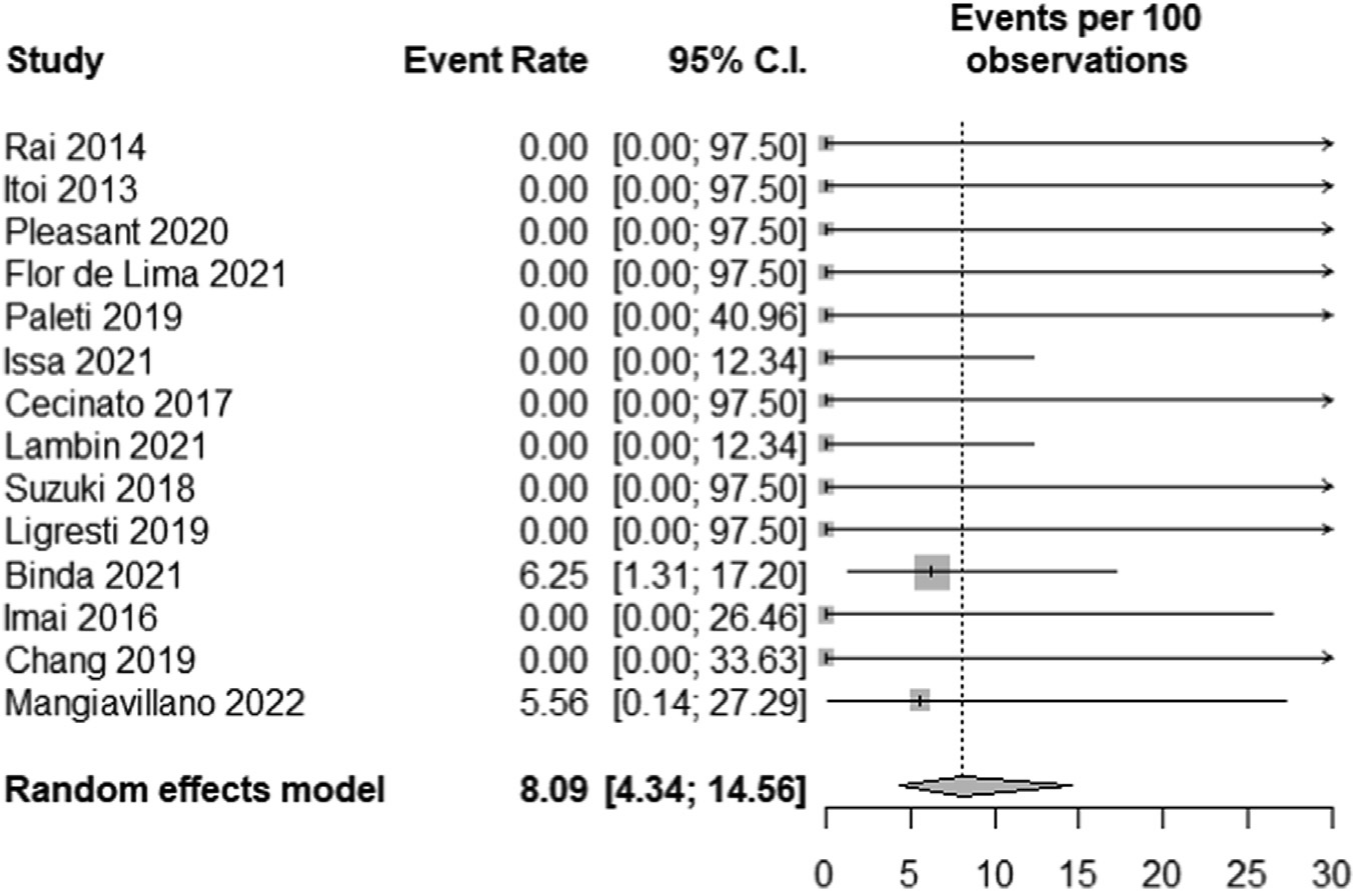
Pooled immediate adverse event rate. *CI*, Confidence interval.

Fourteen studies^3^, ^4^, ^5^^,^^20^, ^21^, ^22^, ^23^, ^24^, ^25^, ^26^, ^27^, ^28^, ^29^, ^30^ reported immediate adverse events with a total of 157 patients included in the meta-analysis. Four patients had immediate adverse events. Two patients had misplacement of the stent intraprocedurally.^20^^,^^30^ Two patients experienced bleeding interprocedurally; 1 patient had this bleeding managed conservatively, and the other was managed with endoscopic hemostasis.^20^ The pooled immediate adverse event rate was 8.09% (95% CI, 4.34-14.56; *I*^2^ = 0) (Fig. 3).

**Figure 4:**
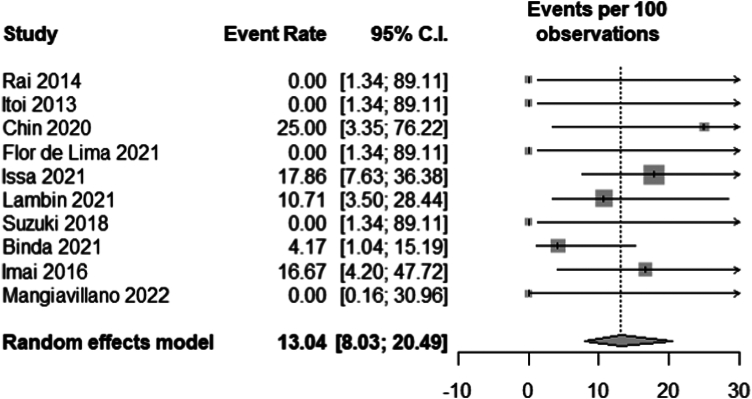
Pooled delayed adverse event rate. *CI*, Confidence interval.

Ten studies^2^, ^3^, ^4^, ^5^^,^^16^^,^^20^^,^^23^, ^24^, ^25^^,^^29^ reported delayed adverse events of EUS-GBD with a total of 149 patients included in the meta-analysis. The delayed adverse events reported were food impaction in the stent (n = 3), stent migration and/or dysfunction (n = 5), bleeding (n = 2), cholangitis (n = 1), peritonitis (n = 1), and unknown adverse events (n = 1). There were no deaths related to the procedure. The pooled delayed adverse event rates were 13.04% (95% CI, 8.03-20.49; *I*^2^ = 0) (Fig. 4).

The pooled adverse event rate (including both immediate and delayed) was 13.81% (95% CI, 9.19-20.23; *I*^2^ = 0).

### Reintervention

**Figure 5:**
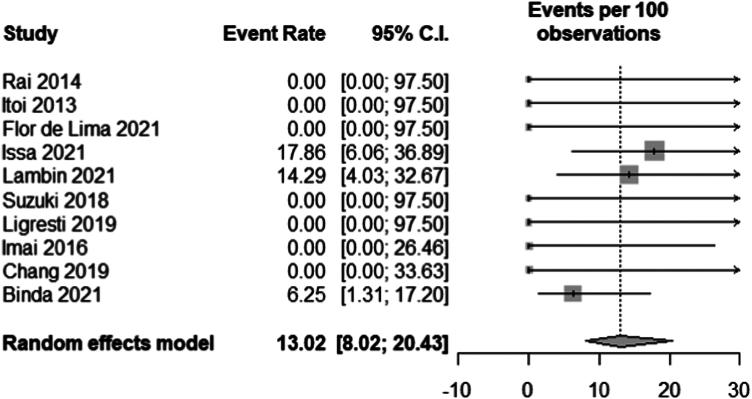
Pooled reintervention rate. *CI*, Confidence interval.

Eleven studies^3^, ^4^, ^5^^,^^20^^,^^22^, ^23^, ^24^, ^25^, ^26^^,^^29^^,^^30^ reported if patients underwent any reintervention, with a total of 130 patients included in the meta-analysis. Twelve patients in 3 studies^3^^,^^20^^,^^25^ underwent reintervention after achieving clinical success. Reintervention was done for food impaction in stent (n = 3), bleeding adverse event (n = 2), stent dysfunction (n = 3), and for unclear reasons (n = 4). The 3 patients who had food impaction in their stent required revision of the stents, which were all further complicated by cholecystitis necessitating re-revision and antibiotics. The 2 patients who had bleeding adverse events were managed endoscopically. One patient had an ulcer with a visible vessel inside the gallbladder managed by use of a hemostatic clip; and the other patient had clots removed from the stent, but no bleeding lesion was found. There was no recurrence of bleeding in either patient, and neither required interventional radiologic/surgical interventions.^3^ One patient had stent dysfunction due to rapid tumor growth that required PTBD.^24^ The other 2 patients with stent dysfunction required insertion of a second lumen-apposing metal stent.^20^ The pooled reintervention rate was 13.02% (95% CI, 8.02-20.43; *I*^2^ = 0) (Fig. 5).

### Sensitivity analysis and publication bias

**Supplementary Figure 2:**
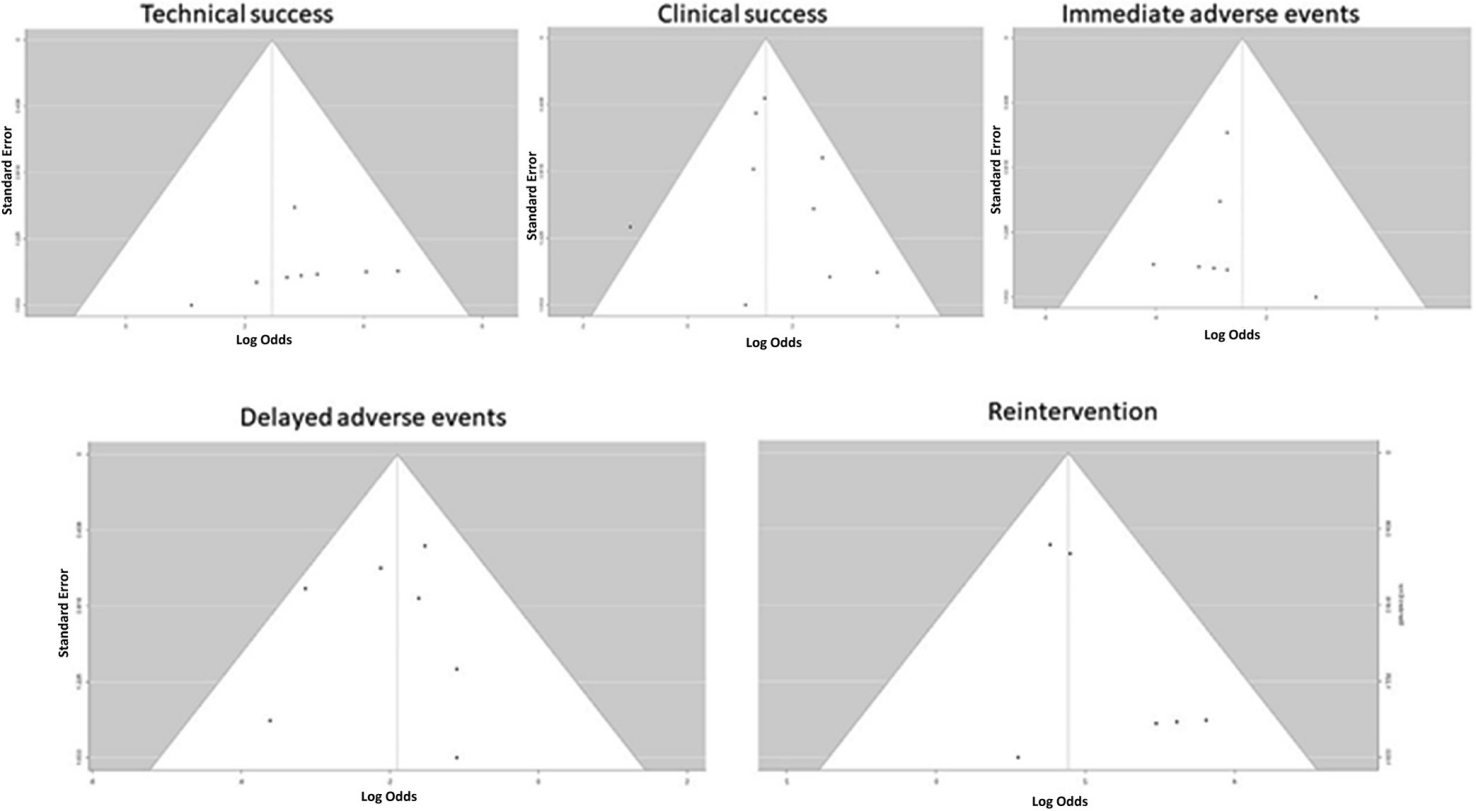
Publication bias.

None of the outcomes had any heterogeneity. Thus, we did not conduct a sensitivity analysis. All outcomes had low probability of publication bias. Funnel plots for all outcomes are reported in Supplementary Figure 2 (available online at www.igiejournal.org).

### Association between type of stent and outcomes

Meta-regression results are presented in Supplementary Table 3 (available online at www.igiejournal.org). The use of LAMSs (compared with self-expandable metal stents) was not associated with different outcomes (*P* values >.05).

## Discussion

This meta-analysis evaluating EUS-GBD as a palliative measure for MDBO found excellent technical success, with effective resolution of the biliary obstruction in >80% of the cases. The technical success rates were comparable between previously reported meta-analyses of ERCP and EUS-BD and our meta-analysis of EUS-GBD (92.66% ERCP vs 92.79% EUS-BD vs 92.10% EUS-GBD). The clinical success of ERCP and EUS-BD was slightly higher, yet still comparable, compared with EUS-GBD (93.20% ERCP vs 93.40% EUS-BD vs 81.62% EUS-GBD). The adverse event rates of EUS-GBD, however, were lower than with ERCP but comparable to EUS-BD (23.85% ERCP vs 12.61% EUS-BD vs 13.81% EUS-GBD). Similarly, the reintervention rates of EUS-GBD were lower than with ERCP but comparable to EUS-BD (28.70% ERCP vs 11.82% EUS-BD vs 13.02% EUS-GBD).^32^ Wang et al^33^ showed that the success rates and adverse events of EUS-BD improved significantly after 2013, which was attributed to greater familiarity of the procedure by the endoscopists and better endoscopic accessories. Furthermore, the volume of EUS-guided procedures performed by the centers in the included studies was not reported. EUS-GBD is a relatively new procedure, first described in 2013, and thus it would be expected that outcomes improve with time as well.

However, the most commonly established intervention, to-date, after ERCP and EUS-BD is percutaneous drainage.^7^ EUS-GBD and PTBD have never been compared in head-to-head studies. As opposed to PTBD, identifying the site of the obstruction relative to the cystic duct is paramount before performing EUS-GBD, which can only be performed if the MDBO is at or distal to the cystic duct. When comparing EUS-GBD with reported outcomes of PTBD, technical and clinical success rates were similar (96.90% PTBD vs 92.10% EUS-GBD and 87.10% PTBD vs 81.62% EUS-GBD, respectively). However, the reintervention rates were considerably higher in PTBD (nearly 50%), mostly due to PTBD malposition/dislodgement, bile leak, and cholangitis.^6^ Quality of life is a very important issue to take into account, particularly when addressing palliative measures. Internal stenting is associated with better quality of life.^34^ Outcomes for internal stenting via PTBD is not well elaborated in the literature.^7^ As such, EUS-GBD, although not objectively assessed, may be associated with better quality of life compared with PTBD.

Another intervention to drain MDBO is EUS-guided hepaticogastrostomy (EUS-HGS).^35^, ^36^, ^37^ In a large study of 110 patients with MDBO, EUS-HGS had a technical and clinical success rate of 100% and 94%, respectively. The adverse event rate was 12%, which included peritonitis, cholangitis, and pseudoaneurysm. Recurrent biliary obstruction was noted in 33% of the cases, which occurred 6.3 months after EUS-HGS was performed. This was managed mainly via the existing EUS-HGS route, except for 1 patient who needed an additional EUS-HGS stent and 2 patients needing PTBD.^35^ The indications of EUS-HGS were similar to the indications of EUS-GBD in our meta-analysis (namely having failed ERCP, ERCP being high risk, or having an altered anatomy). In the studies included in our meta-analysis, 4 studies^4^^,^^21^^,^^23^^,^^28^ mentioned that patients had poor anatomy for an EUS-HGS and thus ultimately underwent an EUS-GBD. This included acute angulation of the needle to the intrahepatic bile ducts^4^ and a lack of significant intrahepatic ductal dilatation.^21^^,^^23^^,^^28^ To date, there has been no head-to-head comparison between EUS-HGS and EUS-GBD in MDBO, which limits us in providing a strong conclusion comparing both interventions. However, the technical and clinical success rates of EUS-HGS were similar to the outcomes we reported in EUS-GBD; in addition, the reintervention rates were relatively higher in EUS-HGS.^35^ Based on the current data, EUS-GBD can be the primary drainage method of MDBO in patients with challenging anatomy (particularly those without significantly dilated intrahepatic bile ducts, rendering EUS-HGS not feasible).

None of the studies reported in literature, to date, included the use of plastic stents in MDBO, which may be due to LAMSs being ideal for preventing bile leaks. However, long-term placement of LAMSs has been associated with multiple adverse events, including bleeding (typically from a pseudoaneurysm) and “buried stent” phenomenon.^38^, ^39^, ^40^ Authors have thus recommended close follow-up with removal of the LAMSs in 3 to 4 weeks to avoid these adverse events.^39^^,^^40^ Most of the studies included in our meta-analysis had a follow-up period of >4 weeks, and there was no report of buried stent phenomenon and only 2 cases of bleeding, which were managed endoscopically. Furthermore, multiple large robust meta-analyses showed that LAMSs had similar adverse events, stent-related adverse events, and bleeding in comparisons of LAMSs versus plastic stents.^41^, ^42^, ^43^ However, all the literature comparing LAMSs and plastic stents is limited to patients with walled-off pancreatic necrosis, rather than an MDBO. Extrapolating the available data on LAMSs and plastic stents, however, we can assume that LAMSs would not have an added risk in patients with MDBO. However, more research is needed to test the validity of this assumption.

The current study has several limitations. First, the results were derived from low-level evidence (case reports and observational studies). However, to date, there are no randomized controlled trials evaluating EUS-GBD in MDBO. Second, we did not incorporate non-English language studies. Third, the meta-analysis is limited by the data provided in the included studies. The severity of the adverse events based on a scoring system, such as the adverse events in GI endoscopy (ie, AGREE) classification or the American Society for Gastrointestinal Endoscopy classification, was not assessed in the studies.^44^^,^^45^ Furthermore, none of the studies stratified the outcomes according to type of malignancy or site/size of the stent. As such, we could not account for these variables in our study. Fourth, there was no statistical comparison evaluating outcomes between EUS-GBD and the other interventions. However, this is again due to the absence of any comparative studies in the literature. Thus, this meta-analysis offers the best report of outcomes of EUS-GBD in MDBO based on the current literature.

In conclusion, EUS-GBD does not seem to be inferior to other proposed interventions in managing MDBO and should be offered to patients. High-quality evidence and comparative studies are needed to formally compare outcomes of EUS-GBD versus those of other interventions and to further outline a patient-tailored strategy to select the most appropriate intervention for patients with MDBO.

## Disclosure

The following author disclosed financial relationships: N. Marya is a consultant for Boston Scientific. All other authors disclosed no financial relationships.

## Figures and Tables

**Supplementary Table 1:** Search Strategy

*#*	*Searches*
*1*	Gallbladder [mesh] OR gallbladder diseases [mesh]
*2*	Gallbladder [tw] OR cholecystitis [tw]
*3*	#1 OR #2
*4*	Drainage [mesh] OR drainage [tw]
*5*	#3 AND #4
*6*	Endoscopy [mesh] OR endosonography [mesh]
*7*	Endoscopy [tw] OR endosonography [tw]
*8*	EUS [tw] OR “EUS-GBD” [tw] OR “endoscopic ultrasound” [tw] OR “endoscopic ultrasonography” [tw]
*9*	#6 OR #7 OR #8
*10*	#5 AND #9
*11*	#5 AND #9 Filters: English
*12*	#5 AND #9 Filters: English, Humans

**Supplementary Table 2:** Quality assessment of included studies

Study (year); location	Selection	Ascertainment	Causality	Reporting	Total	Quality
Case reports						
Rai et al^5^ (2014); India	0	1	1	1	3	Fair
Itoi et al^4^ (2013); United States	0	1	1	1	3	Fair
Pleasant et al^28^ (2020); United States	0	1	0	0	1	Poor
Flor de Lima et al^23^ (2021); Portugal	0	1	1	1	3	Fair
Cecinato et al^21^ (2017); Italy	0	1	0	1	2	Poor
Suzuki et al^29^ (2018); Japan	0	1	1	1	3	Fair
Ligresti et al^26^ (2019); Italy	0	1	0	0	1	Poor

Observational studies	Selection	Outcome			Total	Quality
		Reliably ascertained	Adequate follow-up	All patients accounted at last follow-up		
Chin et al^2^ (2020); New Zealand	1	1	0	0	2	Poor
Paleti et al^27^ (2019); United States	1	1	1	0	3	Fair
Issa et al^3^ (2021); United States	1	1	1	1	4	Good
Lambin et al^25^ (2021); France	1	1	1	0	3	Fair
Binda et al^20^ (2021); Italy	1	1	1	0	3	Fair
Imai et al^24^ (2016); Japan	1	1	0	1	3	Fair
Chang et al^22^ (2019); United States	1	1	1	1	4	Good
Mangiavillano et al^30^ (2022); Italy	1	1	1	1	4	Good

**Supplementary Table 3:** Association between type of stent and outcomes

Outcome	Coefficient (95% CI)	*P* value	*I*^2^
Technical success	0 (–.002 to .003)	.76	0%
Clinical success	–.001 (–.004 to .002)	.54	0%
Immediate adverse events	.003 (–.003 to .003)	.81	3.52%
Delayed adverse events	–.001 (–.004 to .002)	.49	17.13%
Reintervention	.002 (–.001 to .005)	.15	0%

*CI*, confidence interval.
